# Editorial: Metabolic and biomechanical factors in bone fragility: new frontiers in understanding and managing osteoporosis

**DOI:** 10.3389/fendo.2026.1863031

**Published:** 2026-05-05

**Authors:** Kamyar Asadipooya

**Affiliations:** Division of Endocrinology, Diabetes, and Metabolism, Barnstable Brown Diabetes and Obesity Center, University of Kentucky, Lexington, KY, United States

**Keywords:** bone density, bone metabolism, bone quality, fracture, osteoporosis

Skeletal fragility results primarily from loss of bone mass or changing structure that diminish bone quality and strength. Osteoporosis can be classified as primary or secondary, depending on the underlying factors that lead to loss of bone mass, deterioration of microarchitecture or compromised bone metabolism. While postmenopausal osteoporosis is most common, secondary causes can be seen in up to one-third of postmenopausal women and more than half of premenopausal women or men with osteoporosis (1, 2). Although aging is the principal driver of bone loss, metabolic disturbances, systemic illnesses, and organ dysfunction can potentiate the process.

Better understanding of the pathophysiology of bone loss, recognizing the secondary causes of osteoporosis, improving our understanding of the risk factors of fracture, increasing the knowledge of assessing bone fragility or predicting fracture and to end introducing new therapeutic approaches are important research fields. Advancement in this field can be achieved by generating more evidence and reviewing current scientific literature. The Research Topic “Metabolic and Biomechanical Factors in Bone Fragility: New Frontiers in Understanding and Managing Osteoporosis” contains one study protocol, four review and thirteen original research articles. These contributions explore metabolic and inflammatory aspects of bone health, novel methods for detecting bone fragility and emerging treatment options to improve bone strength. The review articles discuss multiple critical aspects of bone fragility: the limitations of bone mineral density (BMD) evaluation by dual energy X-ray absorptiometry (DXA) in secondary osteoporosis (Asadipooya et al.), the correlation between the metabolic effects of steatotic liver disease and bone mass, bone cells function and low-trauma fractures (Li et al.), the immunometabolic characters of macrophages in osteoporotic fractures (Xing et al.), and how muscle-derived myokines affect bone turnover, formation and resorption, and specifically within diabetic condition (Ji et al.). The original research articles explore the method of assessment for bone fragility by using DXA and high-resolution quantitative peripheral computed tomography (HR-pQCT) in patients with familial partial lipodystrophy (Gonçalves Muniz et al.), change in BMD and Quantitative CT (QCT) in patients with primary hyperparathyroidism (Pu et al.), defining β-CTX (beta-C-terminal telopeptide of type I collagen) and estradiol thresholds for predicting perimenopausal bone loss (Feng et al.), and applying available clinical data by means of practical machine learning model to predict osteoporosis in older women (Tang et al.). These manuscripts also investigated the correlation between creatinine clearance and lumbar spine BMD among elderly patients (aged ≥50 years) with low-energy fractures (Su et al.), the linear association of triglyceride-glucose body mass index with all-cause mortality and cardiovascular mortality in 302 patients with osteoporosis (Li et al.), the negative correlation between high-density lipoprotein (HDL) and both β-CTX and P1NP (the procollagen type I N-terminal propeptide) in hospitalized patients with fragility fractures (Zhu et al.), the sigmoidal correlation between neutrophil levels and refracture risk in osteoporotic men (Yuan et al.), and finally, the non-linear S-shaped relationship between triglyceride-glucose index and serum uric acid in patients with osteoporotic fracture (Zhou et al.). They further showed evidence of improved survival in patients with type 2 diabetes mellitus (T2DM) and obesity who received osteoporosis treatment, as demonstrated in a retrospective cohort of 8,788 matched patients (Rouach et al.), comparability of the outcomes (fracture and death) of Zoledronic Acid and Denosumab in 27503 patients with osteoporosis and T2DM (Rouach et al.), and the benefits of organic selenium compound β-trifluoroethoxy dimethyl selenide for organoselenium compound (4aa) in modulating gut microbial composition and function to prevent bone loss in female mice following ovariectomy (Hu et al.). Collectively, these studies provided valuable insights to the landscape of osteoporosis management.

Normally, prevention of fracture is central to effective osteoporosis management. This requires proper tools to accurately predict fracture, particularly when bone damage is related to a systemic illness or organ dysfunction. Therefore, introducing a sensitive method for assessing bone health and judging the probability of fracture based on the risk factors would be important initial steps. Secondly, selecting optimal treatment modalities tailored to the specific mechanisms of bone loss should be more carefully evaluated.

In summary, expanding the scope of osteoporosis diagnosis and management is necessary. Therapeutic approaches should not be restricted to antiosteoporosis medications, but extended to incorporate lifestyle modifications, such as diet and exercise, controlling risk factors, and treating the secondary causes to improve patient outcomes significantly.
